# MMR deficiency is frequent in colorectal carcinomas with diffuse SLFN11 immunostaining: clinicopathologic and molecular study of 31 cases identified among 3,300 tumors

**DOI:** 10.1002/2056-4538.70025

**Published:** 2025-03-19

**Authors:** Maciej Kaczorowski, Małgorzata Chłopek, Ondřej Daum, Kris Ylaya, Tomáš Vaněček, Magdalena Szczepaniak, Karol Krawczyk, Artur Kowalik, Michal Michal, Jerzy Lasota, Markku Miettinen

**Affiliations:** ^1^ Laboratory of Pathology National Cancer Institute Bethesda MD USA; ^2^ Department of Clinical and Experimental Pathology Wrocław Medical University Wrocław Poland; ^3^ Sikl's Institute of Pathology, Faculty of Medicine and Teaching Hospital in Plzen Charles University Plzen Czech Republic; ^4^ Bioptical Laboratory, Ltd. Plzen Czech Republic; ^5^ Department of Molecular Diagnostics Holycross Cancer Center Kielce Poland

**Keywords:** SLFN11, colorectal cancer, mismatch repair proteins, Lynch syndrome, mucinous carcinoma, DNA damaging agents

## Abstract

Schlafen 11 (SLFN11), a regulator of cell fate following DNA injury, sensitizes tumor cells to DNA‐damaging agents. Patients with SLFN11‐positive tumors may benefit from DNA‐damaging chemotherapies. SLFN11 has been studied in different types of cancer including colorectal carcinomas. However, colorectal carcinomas with diffuse positivity (expression in ≥80% of tumor cells) have not been meticulously characterized. SLFN11 immunostaining of tumor microarrays (TMAs) with 3,300 primary CRCs identified 65 (~2.0%) tumors with focal staining (<10% of tumor nuclei positive), 83 (~2.5%) with patchy (≥10% and <80%) and 51 (~1.5%) with diffuse (≥80%) SLFN11 positivity. The latter was confirmed on full sections from donor blocks in 31 (~1%) cases, which were further studied including evaluation of additional immunohistochemical markers, genotyping with targeted DNA sequencing, and assessment of microsatellite instability. SLFN11‐positive carcinomas were mostly (21/31, 68%) right‐sided tumors with a female predominance (21/31, 68%) and median age of 67 years. Eighteen of 31 (58%) contained areas of mucinous differentiation. Deficiency of mismatch repair proteins was detected in 65% (20/31) of SLFN11‐positive carcinomas. Moreover, *MLH1* (*n* = 2), *MSH2*, *MSH6*, and *PMS2* germline mutations were identified in 25% (5/20) of patients with mismatch repair deficient tumors. *BRAF* p.V600E mutation was found in 45% (9/20) of mismatch repair deficient, but only 1 of 11 proficient tumors. Colorectal carcinomas with diffuse SLFN11 positivity were often mismatch repair deficient tumors with their typical clinical, morphological, and molecular characteristics.

## Introduction

Schlafen 11 (SLFN11) is a DNA helicase which controls cellular response to replicative stress [1, 2]. Upon DNA injury, SLFN11 permanently stalls replication forks and induces apoptosis [3]. Therefore, its expression has been increasingly recognized as a biomarker of tumor sensitivity to DNA‐targeting therapies, including platins, hydroxyurea, nucleoside analogs, topoisomerase inhibitors, and poly(ADP‐ribose) polymerase inhibitors [4, 5, 6, 7, 8]. Tumors lacking SLFN11 may be resistant to those agents and require alternative treatments [4, 7, 9, 10, 11, 12]. Thus, the analysis of SLFN11 expression is among inclusion criteria for several ongoing clinical trials (clinicaltrials.gov), and it may eventually become a part of routine clinical practice.

Colorectal carcinoma (CRC) is the third most common and second most deadly cancer worldwide with almost 2 million new cases diagnosed in 2022 [13]. Biomarker‐informed, personalized treatment may improve survival of CRC patients. Variable expression of SLFN11 was reported in primary (~5%) and metastatic (~10%) CRCs [14] and linked to sensitivity to platinum derivatives [15].

This study aimed to identify CRCs with diffuse expression of SLFN11. Such tumors have not been systematically characterized for their clinicopathological, immunophenotypic, and molecular genetic features.

## Materials and methods

### Tissue samples and immunohistochemical screening for SLFN11‐positive cases

This study was completed under the Office of Human Subject Research Exemption (https://www.hhs.gov) with clinically annotated de‐identified specimens. Previously characterized 3,300 primary CRCs [16] from 124 tissue microarrays were screened for SLFN11 expression using immunohistochemistry (IHC). Leica Bond Max automation platform (Leica Biosystems, Bannockburn, IL, USA) and anti‐SLFN11 D‐2 antibody (mouse monoclonal, catalog number: sc‐515,071; Santa Cruz Biotechnology, Dallas, TX, USA) was employed as previously reported [14]. Diffusely positive (≥80% of positive nuclei) tissue microarray (TMA) spots were validated with full tissue section of donor blocks. Tumors with diffuse SLFN11 expression were selected and 2nd generation TMAs (*n* = 2) comprising validated cases were constructed for further IHC evaluation.

### Characteristics of CRCs with diffuse SLFN11 expression

#### Clinicopathologic data, histopathology, and IHC

pTNM categories were compliant with the 8th Edition of the American Joint Committee on Cancer staging system [17]. Tumor histopathologic classification was done following the WHO Classification of Tumours of the Digestive System, 5th edition [18].

Expression of caudal type homeobox 2 (CDX2), cytokeratin 7 (CK7), cytokeratin 20 (CK20), beta‐catenin (β‐catenin), and tumor protein p53 (p53) was evaluated using Ventana BenchMark Ultra (Ventana Medical Systems, Tucson, AZ, USA) IHC. Staining was graded as negative, focal (<10% of tumor cells), patchy (≥10% and <80% of tumor cells) and diffuse (≥80% tumor cells). p53 immunolabeling was categorized as normal/wild type (WT) (scattered cells with variable intensity of nuclear staining), negative (complete loss of nuclear expression), or diffuse/overexpressed (strong staining in ≥80% of tumor nuclei).

#### DNA and RNA extraction

Genomic DNA was isolated from formalin‐fixed paraffin‐embedded (FFPE) tumor tissues of all analyzed CRCs. In five cases, non‐neoplastic DNA or RNA was obtained from peripheral blood samples or extracted from adjacent non‐neoplastic tissues in three cases.

#### Targeted DNA next‐generation sequencing

Tumor DNA samples were studied using AmpliSeq™ Cancer Hotspot Panel v2 Kit and Ion Torrent® next‐generation sequencing platform (Thermo‐Fisher Scientific, Waltham, MA, USA). The assay evaluated genomic status of 50 oncogenes and tumor suppressors commonly mutated in cancer.

#### Analysis of mismatch repair and microsatellite instability status

In all cases, immunohistochemical expression of MutS Protein Homolog 2 (MSH2), MutS Protein Homolog 2 (MSH6), MutL Protein Homolog 1 (MLH1), and Postmeiotic Segregation Increased 2 (PMS2) was evaluated with Ventana BenchMark Ultra platform.

*MLH1* promoter hypermethylation, determined by the methylation‐specific PCR targeting promoter sequences following the bisulfite conversion with EZ DNA Methylation‐Gold Kit (Zymo Research, Burlington, ON, USA) [19] was analyzed in 11 cases.

An instability of mononucleotide markers, *BAT‐25*, *BAT‐26*, *NR‐21*, *NR‐24*, and *MONO‐27* was assessed using Droplet Digital™ PCR (ddPCR™) MSI assay (Bio‐RAD, Hercules, CA, USA) in five cases including four tumors screened for MLH1, MSH2, MSH6, PMS2, and POLE mutations using a custom gene panel created with the Ion Ampliseq Designer for Ion Torrent NGS platform (Thermo‐Fisher Scientific).

### Normal tissue

*MLH1*, *MSH2*, and *MSH6* coding and exon‐intron junction sequences and *PMS2* coding sequences were evaluated using the Sanger method following PCR or RT‐PCR amplification of DNA or RNA extracted from peripheral blood samples (*n* = 5). In addition, three DNA samples from non‐neoplastic tissues adjacent to *MSH2* (*n* = 1), *MSH6* (*n* = 1), and *MLH1* (*n* = 1) mutant tumors (identified by a custom gene NGS panel) were germline tested using mutation‐specific PCR amplification and Sanger sequencing.

In five cases, the selective adaptor ligation selective amplification methylation sensitive‐multiplex ligation probe amplification (SALSA® MS‐MLPA®) kits P003, P072, P008, and ME011 (MRC‐Holland, Amsterdam, The Netherlands) were used to detect large genomic aberrations such as deletions and duplications in *MLH1*, *MSH2*, *MSH6*, and *PMS2*. The PCR amplification products were separated by capillary electrophoresis using ABI 3130XL Genetic Analyzer (Applied Biosystems, Waltham, MA, USA), and the output data were analyzed with ‘Coffalyser.net’ software (MRC‐Holland).

Details of antibodies and immunohistochemical protocols, molecular genetic methods including DNA and RNA extraction, Sanger sequencing and NGS, ddPCR™, and SALSA® MS‐MLPA® and study the deficiency of mismatch repair (dMMR) and MSI are provided in Supplementary materials and methods.

## Results

### Identification of CRCs with diffuse SLFN11 immunopositivity

**Figure 1:**
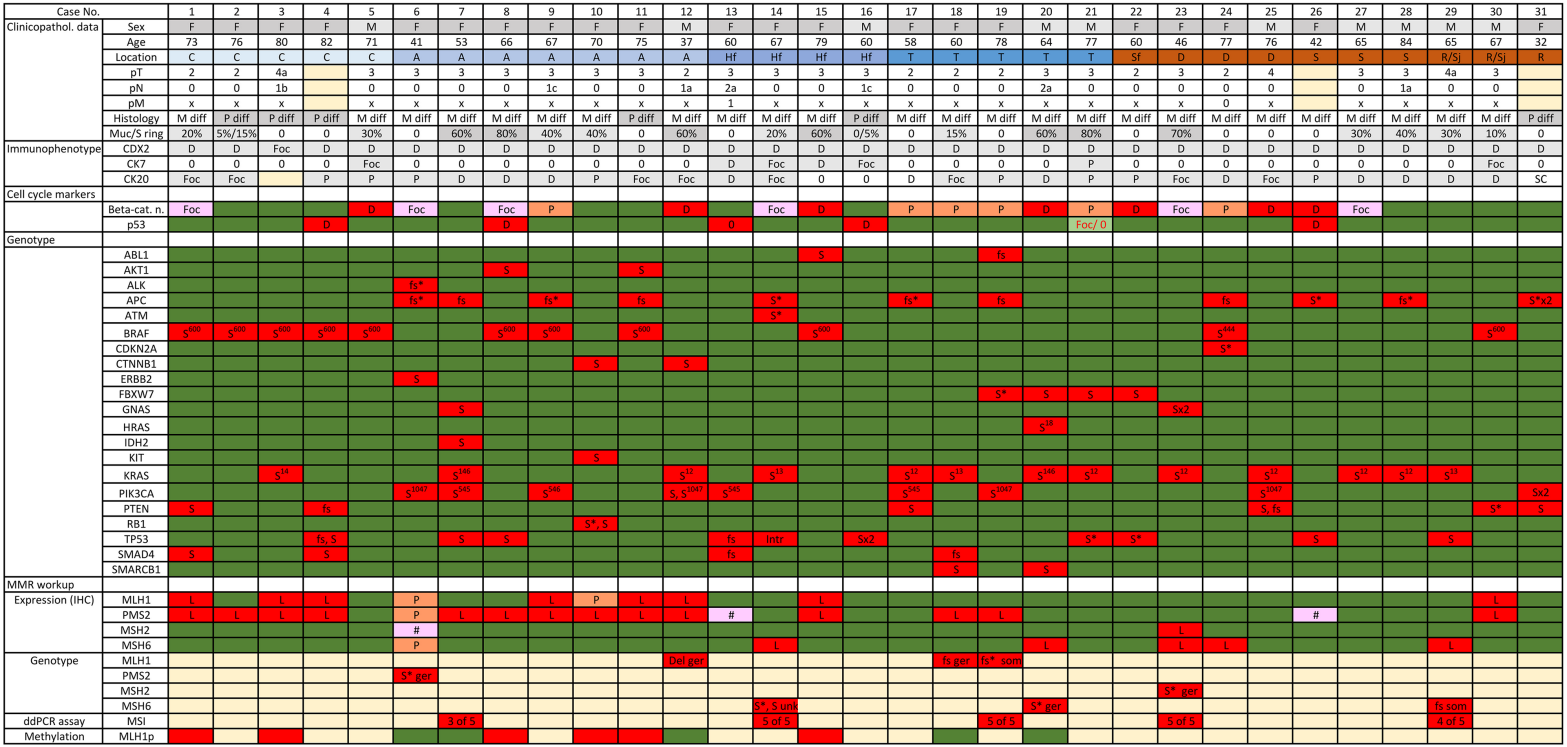
Clinicopathologic and molecular genetic characteristics of 31 CRCs with diffuse SLFN11 expression. Abbreviations (from the first to the last row left to right in consecutive order) are as follows: F, female; M, male; C, cecum; A, ascending, Hf, hepatic flexure; T, transverse; Sf, splenic flexure; D, descending; S, sigmoid; R/Sj, rectosigmoid junction; R, rectum; M diff, moderately differentiated; P diff, poorly differentiated; Muc, mucinous; S.ring, signet ring; 0, not present/negative/lack of expression; D, diffuse; Foc, focal; P, patchy; SC, scattered cells; Foc/0, indicates heterogeneity; Beta‐cat., β‐catenin; n., nuclear; S, substitution (missense mutation); fs, frameshift; *, STOP codon; superscript number, codon number; L, lost; #, intraglandular heterogeneity; Del, deletion; ger, germline; som, somatic; unk, unknown (germline versus somatic variant). Colors: blue – right colon; brown – left colon; yellow – not available; green – normal expression and WT; red – pathologic changes; orange and pink – possible pathologic changes. Dark and light gray mark female and male, respectively. Also, shades of gray are used to mark morphological differences. ddPCR assay: numbers indicate altered MSI markers.

**Figure 2:**
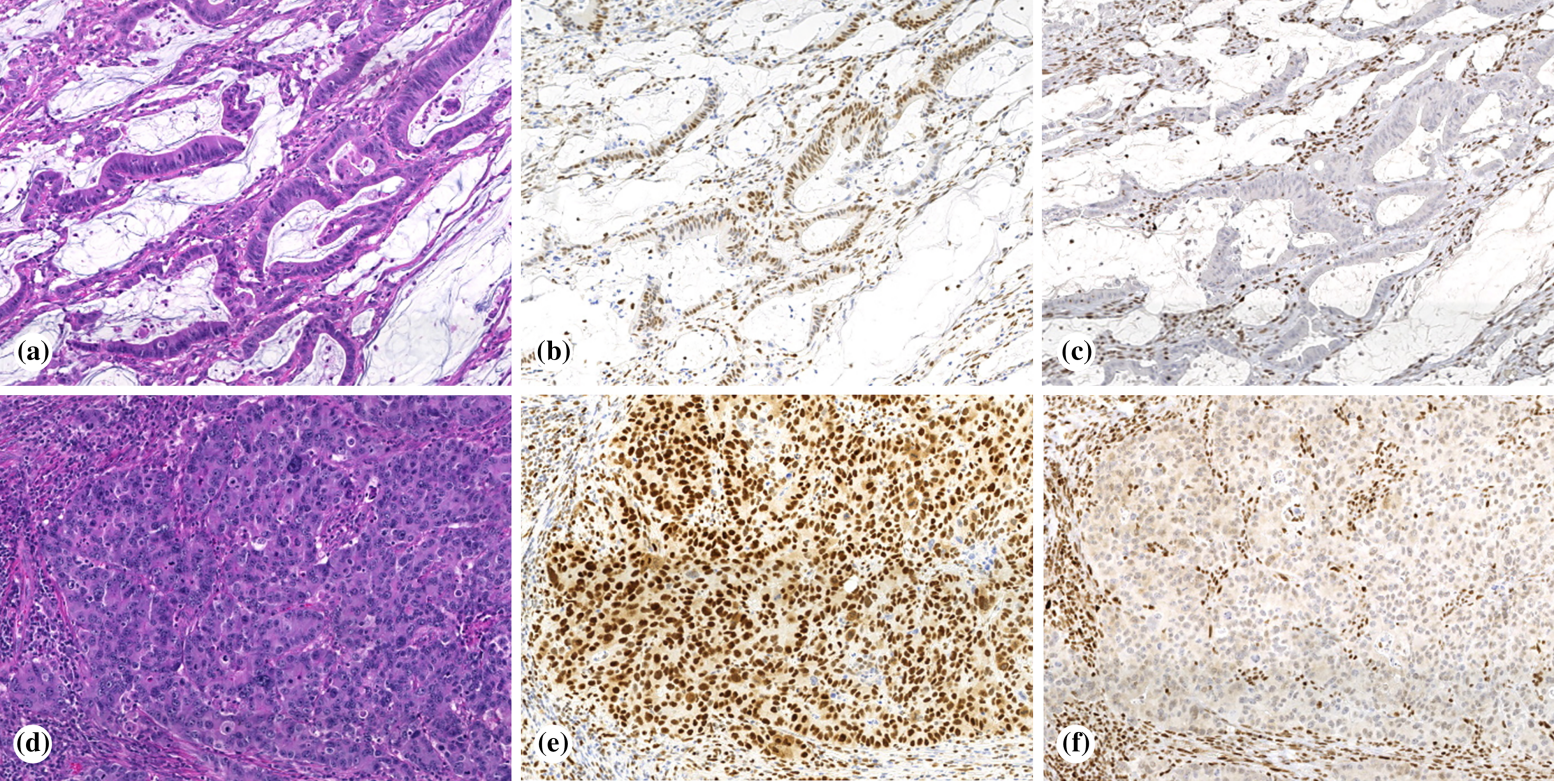
Representative images. (A–C) Adenocarcinoma of the colonic hepatic flexure (case number 14) with mucinous differentiation (A, ×100) showing diffuse SLFN11 staining (B, ×100) and MSH6 loss (C, ×100) by immunohistochemistry; this tumor had mutations in *MSH6* and *KRAS* genes. (D–F) *BRAF* V600E‐mutated carcinoma of the cecum (case number 4) (D, ×100) with SLFN11 positivity (E, ×100) and MLH1 loss (F, ×100).

SLFN11 immunostaining of TMAs with 3,300 primary CRCs identified 65 (~2.0%) tumors with focal staining (<10% of tumor nuclei positive), 83 (~2.5%) with patchy (≥10% and <80%), and 51 (~1.5%) with diffuse (≥80%) SLFN11 positivity. The latter was confirmed on full sections from donor blocks in 31 cases, which were further studied including evaluation of additional immunohistochemical markers, genotyping with targeted DNA sequencing, and assessment of microsatellite instability. Demographic, clinicopathologic, immunohistochemical, and molecular features of CRCs with diffuse SLFN11 expression are summarized in Figure 1. Filtering and sorting of the tumors by different variables included in Figure 1 is possible in supplementary material, Table S1. Representative photomicrographs are shown in Figure 2.

### Demographic and clinicopathologic data

The cohort consisted of 21 females (68%) and 10 males. The patient age ranged from 32 to 84 with a median age of 67 years for females and 66 years for males. A majority (68%) of SLFN11‐positive CRCs occurred on the right side of the colon. All but one tumor with a strong SLFN11 immunoreactivity were right‐sided. The female to male ratio was 3:1 and 1:1 for right‐ and left‐sided tumors, respectively. The pTNM staging was available in 28 cases, although the status of metastatic disease was unknown in all but two patients. The most common was stage pT3 reported in 61% (17/28) of CRCs followed by pT2 (*n* = 8) and pT4 (*n* = 3). Seven (25%) of 28 patients had local metastases at the surgery.

### Histopathological features, immunophenotype, and genotype

Eighteen (58%) of 31 CRCs contained areas of mucinous differentiation ranging from 5% to 80% of total tumor area. Seven cases were bona fide mucinous adenocarcinomas with extracellular mucin comprising >50% of tumor area. Two CRCs contained areas with signet ring cell morphology. Six tumors were poorly differentiated carcinomas with a solid sheet pattern.

Immunohistochemically, all tumors that expressed CDX2 and 28/31 were CK20‐positive including 11 cases with diffuse staining. CK7 positivity was found in seven tumors, diffuse in two of them. Of 31 tumors, 25 had WT and 5 had aberrant (4 overexpressed and 1 null) pattern of p53 immunoreactivity. One tumor contained subclones with WT and null p53 phenotypes. Nuclear β‐catenin staining was present in 18/31 tumors and in 7 cases diffuse.

Gain‐of‐function substitutions in *KRAS* codons 12, 13, and 146 and *BRAF* V600E mutation were identified in 38.7% (12/31) and 32.3% (10/31) of cases. Nine tumors (29%) harbored *PIK3CA* mutations mostly affecting common hotspots, codon 1,047 (*n* = 4) and 545 (*n* = 3). Oncogenic *PIK3CA* activation was the major driver in 4 *KRAS*/*BRAF* WT tumors. Mutations in *APC* and *TP53* tumor suppressors were detected in 35.5% (11/31) and 29% (9/31) of cases and were mutually exclusive with a few exceptions (*n* = 3). Detailed results of targeted NGS sequencing of tumor DNA are provided in supplementary material, Table S2.

### Mismatch repair and microsatellite instability status

dMMR workup is summarized in Figure 1. IHC testing of MMR proteins identified losses of single or multiple proteins in 20/31 (65%) tumors. Hypermethylation of *MLH1* promoter was documented in 6 (54.5%) of 11 dMMR cases. In four of five *MLH1* non‐hypermethylated tumors germline mutations affecting *MLH1* (*n* = 2), *PMS2* and *MSH6* were detected by Sanger sequencing. Subsequent NGS of four dMMR tumors not included in *MLH1* hypermethylation analysis detected *MSH6*, *MLH1* somatic, *MSH2* germline, and *MSH6* undetermined mutation. Moreover, MSI was identified in these four cases and *MLH1* non‐hypermethylated tumor (case number 14) by ddPCR MSI assay.

A detailed description of MMR gene mutations is provided in supplementary material, Table S3.

### Deficient MMR versus proficient MMR CRCs

dMMR tumors occurred predominantly in female patients (16/20, 80%), whereas distribution of mismatch proficient (pMMR) cases was balanced between females (*n* = 5) and males (*n* = 6). Also, the proportion of right‐sided location was higher in dMMR (16/20, 80%) compared with pMMR (5/11, 45%) CRCs. Median age at diagnosis was overall higher in cases with dMMR (67) than pMMR (60) tumors. This difference was even more prominent in female patients (71.5 and 58 years, respectively). pTNM data were available for 19/20 dMMR and 9/11 pMMR CRCs. In the former, 13 (68%) tumors were staged pT3 or pT4 and 4 (21%) were pN(+). Among pMMR cancers, seven (78%) were staged pT3 or pT4 and three (33%) were pN(+). Mucinous differentiation was more frequent in dMMR (14/20, including 6 mucinous carcinomas) than pMMR (4/11, including 1 mucinous carcinoma) CRCs.

*BRAF* p.V600E was the most common (9/20, 45%) oncogenic driver mutation in dMMR tumors, whereas *KRAS* (7/20, 35%) and *PIK3CA* (3/20, 15%) were less frequently mutated. Conversely, in pMMR tumors, mutations of the latter two genes were more common (5/11, 45% and 3/11, 27%, respectively) while *BRAF* p.V600E was rare (1/11, 9%).

## Discussion

SLFN11 expression is emerging as a clinically valuable predictive biomarker for optimization of cancer treatment. Several clinical trials are currently investigating new treatment strategies in SLFN11‐positive tumors (clinicaltrials.gov). SLFN11 expression suppressed CRC growth *in vitro* and *in vivo* and sensitized cancer cells to cisplatin [15]. Similarly, elevated SLFN11 expression in CRC cells promoted the efficacy of a topoisomerase inhibitor irinotecan [20]. Overall, analysis of SLFN11 may become useful for clinical decision‐making in CRC patients. However, data on the SLFN11 expression in CRC are incomplete. One study reported high SLFN11 immunoreactivity in 17% (40/236) of analyzed cases, but the staining was cytoplasmic [21], a pattern deemed non‐specific [14, 22]. Another study did not detect any positivity in 39 cases of CRCs analyzed [22]. Our previous immunohistochemical screening of SLFN11 expression in various cancers identified variably positive staining in ~5% of primary CRCs [14]. The current study investigated SLFN11 expression in archival CRC specimens and found that tumors with diffuse immunoreactivity (~1%, 31/3,300) were highly enriched with dMMR tumors. These findings corroborate a previously reported trend associating *SLFN11* mRNA levels and MSI status in CRC [23].

*BRAF* p.V600E mutation was remarkably common (9/20, 45%) in dMMR SLFN11‐positive CRCs. This mutation occurs in ~10% of all CRCs but is significantly enriched in dMMR tumors [24, 25]. Only one of 11 (9%) pMMR SLFN11‐positive tumors harbored *BRAF* mutation. Although a functional link between oncogenic BRAF signaling and SLFN11 expression cannot be excluded, increased representation of *BRAF* mutants among SLFN11‐positive CRCs results from enrichment in dMMR tumors.

One study found the *SLFN11* gene to be methylated in 55% of CRCs, which correlated with *SLFN11* mRNA levels [15]. *SLFN11* methylation was not associated with background clinicopathological features or *BRAF* and *KRAS* mutations; however, it predicted worse overall and relapse‐free survival [15].

Tumors with defective MMR machinery share a hypermutated phenotype that promotes generation of immunogenic neoantigens [26]. Therefore, dMMR/MSI‐H CRCs can be targeted by immune checkpoint blockade [27, 28]; however, ~30% of cases are unresponsive [28, 29]. Development of strategies to overcome this resistance is of paramount importance. Also, 5‐fluorouracil‐based chemotherapy, a standard of care in advanced pMMR CRCs [30], is not effective in dMMR tumors [31]. In this context, SLFN11‐positive dMMR CRCs may benefit from combining checkpoint inhibitors with DNA‐targeting drugs.

The nature of the link between MSI/dMMR and SLFN11 expression is unclear. Immune activation is a hallmark feature of MSI/dMMR CRC [32, 33, 34]. Tumor‐infiltrating lymphocytes and NK cells are robust sources of interferon (IFN)‐γ which is crucial for effective antitumor immunity [35]. Previous studies demonstrated that the *SLFN11* gene is inducible by IFN signaling, which may explain the high representation of SLFN11‐positive CRCs among immunologically hot dMMR tumors [36, 37]. Interestingly, SLFN11 expression can be driven by gain‐of‐function JAK mutations [38] and activation of the JAK–STAT pathway was linked with hyperresponsiveness to IFN‐γ in an MSI‐H CRC cell line [39]. It will be of interest to analyze the relationship between SLFN11 and MMR/MSI status in other tumors. Interrelations between the expression of SLFN11 and the tumor microenvironment are more complex. Rather than a by‐effect of an IFN‐γ‐rich milieu, SLFN11 itself actively regulated the immune landscape and was associated with the efficacy of anti‐PD‐1 therapy in hepatocellular carcinoma [40].

Future research should establish a clinically relevant threshold for SLFN11 positivity in CRC. Diffuse SLFN11 expression was found in ~1% of cases in the current study. Considering the prevalence of CRC, even such a low percentage translates to a considerable number of patients who may potentially receive a more effective, biomarker‐informed therapy. This group may be much wider if non‐diffuse SLFN11 expression is found sufficient to determine improved treatment response. Tumors with any degree of SLFN11 immunopositivity constituted ~6% of all CRCs in this investigation.

In conclusion, SLFN11 is extensively expressed in a minor subset of CRCs. Among tumors with diffuse SLFN11 immunoreactivity there is a high representation of dMMR cases with their typical clinical, morphological, and molecular features.

## Author contributions statement

MK and JL: study design; MK, MC, KY, TV, MS, KK: carrying out experiments; MK, OD, MiM, JL, MaM: data collection; MK, MC, AK, JL: data analysis and interpretation; MK and JL: literature search; MK and JL: generation of figures; MK and JL: drafting of the manuscript; JL and MaM: supervision. All authors approved the submitted version of the manuscript.

## Supporting information


Supplementary materials and methods.

**Table S1.** Clinicopathological, immunophenotypic, and molecular characteristics of colorectal cancers with diffuse SLFN11 expression

**Table S2.** Results of targeted NGS sequencing of tumor DNA from colorectal cancers with diffuse SLFN11 expression

**Table S3.** Description of MMR gene mutations identified in colorectal cancers with diffuse SLFN11 expression

## Data Availability

The datasets generated and/or analyzed in this study are available from the corresponding author upon reasonable request.

## References

[1] Murai J , Thomas A , Miettinen M , *et al*. Schlafen 11 (SLFN11), a restriction factor for replicative stress induced by DNA‐targeting anti‐cancer therapies. Pharmacol Ther 2019; 201: 94–102. 31128155 10.1016/j.pharmthera.2019.05.009 PMC6708787

[2] Jo U , Pommier Y . Structural, molecular, and functional insights into Schlafen proteins. Exp Mol Med 2022; 54: 730–738. 35768579 10.1038/s12276-022-00794-0 PMC9256597

[3] Murai J , Tang SW , Leo E , *et al*. SLFN11 blocks stressed replication forks independently of ATR. Mol Cell 2018; 69: 371–384.e6. 29395061 10.1016/j.molcel.2018.01.012 PMC5802881

[4] Winkler C , Armenia J , Jones GN , *et al*. SLFN11 informs on standard of care and novel treatments in a wide range of cancer models. Br J Cancer 2020; 124: 951–962. 33339894 10.1038/s41416-020-01199-4 PMC7921667

[5] Willis SE , Winkler C , Roudier MP , *et al*. Retrospective analysis of Schlafen11 (SLFN11) to predict the outcomes to therapies affecting the DNA damage response. Br J Cancer 2021; 125: 1666–1676. 34663950 10.1038/s41416-021-01560-1 PMC8651811

[6] Lok BH , Gardner EE , Schneeberger VE , *et al*. PARP inhibitor activity correlates with slfn11 expression and demonstrates synergy with temozolomide in small cell lung cancer. Clin Cancer Res 2017; 23: 523–535. 27440269 10.1158/1078-0432.CCR-16-1040 PMC5241177

[7] Taniyama D , Sakamoto N , Takashima T , *et al*. Prognostic impact of Schlafen 11 in bladder cancer patients treated with platinum‐based chemotherapy. Cancer Sci 2022; 113: 784–795. 34808009 10.1111/cas.15207 PMC8819307

[8] Hamada S , Kano S , Murai J , *et al*. Schlafen family member 11 indicates favorable prognosis of patients with head and neck cancer following platinum‐based chemoradiotherapy. Front Oncol 2023; 12: 978875. 36741698 10.3389/fonc.2022.978875 PMC9892834

[9] Tang SW , Thomas A , Murai J , *et al*. Overcoming resistance to DNA‐targeted agents by epigenetic activation of schlafen 11 (SLFN11) expression with class I histone deacetylase inhibitors. Clin Cancer Res 2018; 24: 1944–1953. 29391350 10.1158/1078-0432.CCR-17-0443 PMC5899656

[10] Nogales V , Reinhold WC , Varma S , *et al*. Epigenetic inactivation of the putative DNA/RNA helicase SLFN11 in human cancer confers resistance to platinum drugs. Oncotarget 2015; 7: 3084–3097. 10.18632/oncotarget.6413 PMC4823092 26625211

[11] Jo U , Murai Y , Chakka S , *et al*. SLFN11 promotes CDT1 degradation by CUL4 in response to replicative DNA damage, while its absence leads to synthetic lethality with ATR/CHK1 inhibitors. Proc Natl Acad Sci U S A 2021; 118: e2015654118. 33536335 10.1073/pnas.2015654118 PMC8017720

[12] Murai J , Feng Y , Yu GK , *et al*. Resistance to PARP inhibitors by SLFN11 inactivation can be overcome by ATR inhibition. Oncotarget 2016; 7: 76534–76550. 27708213 10.18632/oncotarget.12266 PMC5340226

[13] Bray F , Laversanne M , Sung H , *et al*. Global cancer statistics 2022: GLOBOCAN estimates of incidence and mortality worldwide for 36 cancers in 185 countries. CA Cancer J Clin 2024; 74: 229–263. 38572751 10.3322/caac.21834

[14] Kaczorowski M , Ylaya K , Chłopek M , *et al*. Immunohistochemical evaluation of Schlafen 11 (SLFN11) expression in cancer in the search of biomarker‐informed treatment targets: a study of 127 entities represented by 6658 tumors. Am J Surg Pathol 2024; 48: 1512–1521. 10.1097/PAS.0000000000002299 39185596

[15] He T , Zhang M , Zheng R , *et al*. Methylation of SLFN11 is a marker of poor prognosis and cisplatin resistance in colorectal cancer. Epigenomics 2017; 9: 849–862. 28403629 10.2217/epi-2017-0019

[16] Lasota J , Chłopek M , Wasąg B , *et al*. Colorectal adenocarcinomas harboring ALK fusion genes: a clinicopathologic and molecular genetic study of 12 cases and review of the literature. Am J Surg Pathol 2020; 44: 1224–1234. 32804454 10.1097/PAS.0000000000001512 PMC9440614

[17] Weiser MR . AJCC 8th edition: colorectal cancer. Ann Surg Oncol 2018; 25: 1454–1455. 29616422 10.1245/s10434-018-6462-1

[18] Nagtegaal ID , Odze RD , Klimstra D , *et al*. The 2019 WHO classification of tumours of the digestive system. Histopathology 2020; 76: 182–188. 31433515 10.1111/his.13975 PMC7003895

[19] Chan AOO , Broaddus RR , Houlihan PS , *et al*. CpG island methylation in aberrant crypt foci of the colorectum. Am J Pathol 2002; 160: 1823–1830. 12000733 10.1016/S0002-9440(10)61128-5 PMC1850869

[20] Tian L , Song S , Liu X , *et al*. Schlafen‐11 sensitizes colorectal carcinoma cells to irinotecan. Anticancer Drugs 2014; 25: 1175–1181. 25089570 10.1097/CAD.0000000000000151

[21] Deng Y , Cai Y , Huang Y , *et al*. High SLFN11 expression predicts better survival for patients with KRAS exon 2 wild type colorectal cancer after treated with adjuvant oxaliplatin‐based treatment. BMC Cancer 2015; 15: 1–7. 26525741 10.1186/s12885-015-1840-6 PMC4631086

[22] Takashima T , Sakamoto N , Murai J , *et al*. Immunohistochemical analysis of SLFN11 expression uncovers potential non‐responders to DNA‐damaging agents overlooked by tissue RNA‐seq. Virchows Arch 2021; 478: 569–579. 32474729 10.1007/s00428-020-02840-6 PMC9175511

[23] Ballestrero A , Bedognetti D , Ferraioli D , *et al*. Report on the first SLFN11 monothematic workshop: from function to role as a biomarker in cancer. J Transl Med 2017; 15: 199. 28969705 10.1186/s12967-017-1296-3 PMC5625715

[24] Rajagopalan H , Bardelli A , Lengauer C , *et al*. Tumorigenesis: RAF/RAS oncogenes and mismatch‐repair status. Nature 2002; 418: 934. 12198537 10.1038/418934a

[25] French AJ , Sargent DJ , Burgart LJ , *et al*. Prognostic significance of defective mismatch repair and BRAF V600E in patients with colon cancer. Clin Cancer Res 2008; 14: 3408–3415. 18519771 10.1158/1078-0432.CCR-07-1489 PMC2674786

[26] Muzny DM , Bainbridge MN , Chang K , *et al*. Comprehensive molecular characterization of human colon and rectal cancer. Nature 2012; 487: 330–337. 22810696 10.1038/nature11252 PMC3401966

[27] Le DT , Durham JN , Smith KN , *et al*. Mismatch repair deficiency predicts response of solid tumors to PD‐1 blockade. Science 2017; 357: 409–413. 28596308 10.1126/science.aan6733 PMC5576142

[28] Overman MJ , McDermott R , Leach JL , *et al*. Nivolumab in patients with metastatic DNA mismatch repair‐deficient or microsatellite instability‐high colorectal cancer (CheckMate 142): an open‐label, multicentre, phase 2 study. Lancet Oncol 2017; 18: 1182–1191. 28734759 10.1016/S1470-2045(17)30422-9 PMC6207072

[29] André T , Shiu K‐K , Kim TW , *et al*. Pembrolizumab in microsatellite‐instability–high advanced colorectal cancer. N Engl J Med 2020; 383: 2207–2218. 33264544 10.1056/NEJMoa2017699

[30] Morris VK , Kennedy EB , Baxter NN , *et al*. Treatment of metastatic colorectal cancer: ASCO guideline. J Clin Oncol 2023; 41: 678–700. 36252154 10.1200/JCO.22.01690 PMC10506310

[31] Sargent DJ , Marsoni S , Monges G , *et al*. Defective mismatch repair as a predictive marker for lack of efficacy of fluorouracil‐based adjuvant therapy in colon cancer. J Clin Oncol 2010; 28: 3219–3226. 20498393 10.1200/JCO.2009.27.1825 PMC2903323

[32] Llosa NJ , Cruise M , Tam A , *et al*. The vigorous immune microenvironment of microsatellite instable colon cancer is balanced by multiple counter‐inhibitory checkpoints. Cancer Discov 2015; 5: 43–51. 25358689 10.1158/2159-8290.CD-14-0863 PMC4293246

[33] Lin A , Zhang J , Luo P . Crosstalk between the MSI status and tumor microenvironment in colorectal cancer. Front Immunol 2020; 11: 552467. 10.3389/fimmu.2020.02039 PMC7435056 32903444

[34] Phillips SM , Banerjea A , Feakins R , *et al*. Tumour‐infiltrating lymphocytes in colorectal cancer with microsatellite instability are activated and cytotoxic. Br J Surg 2004; 91: 469–475. 15048750 10.1002/bjs.4472

[35] Du W , Frankel TL , Green M , *et al*. IFNγ signaling integrity in colorectal cancer immunity and immunotherapy. Cell Mol Immunol 2021; 19: 23–32. 34385592 10.1038/s41423-021-00735-3 PMC8752802

[36] Borrego AR , Corona‐Ayala C , Salvador JC , *et al*. Gene expression regulation of the type I interferon‐induced protein Schlafen 11. FASEB J 2020; 34: 1.

[37] Mezzadra R , De Bruijn M , Jae LT , *et al*. SLFN11 can sensitize tumor cells towards IFN‐γ‐mediated T cell killing. PLoS One 2019; 14: e0212053. 30753225 10.1371/journal.pone.0212053 PMC6372190

[38] Murai Y , Jo U , Murai J , *et al*. Schlafen 11 expression in human acute leukemia cells with gain‐of‐function mutations in the interferon‐JAK signaling pathway. iScience 2021; 24: 103173. 34693224 10.1016/j.isci.2021.103173 PMC8517841

[39] Yuan W , Deng D , Jiang H , *et al*. Hyperresponsiveness to interferon gamma exposure as a response mechanism to anti‐PD‐1 therapy in microsatellite instability colorectal cancer. Cancer Immunol Immunother 2019; 68: 257–268. 30406373 10.1007/s00262-018-2270-5 PMC11028335

[40] Zhou C , Weng J , Liu C , *et al*. Disruption of SLFN11 deficiency‐induced CCL2 signaling and macrophage M2 polarization potentiates anti‐PD‐1 therapy efficacy in hepatocellular carcinoma. Gastroenterology 2023; 164: 1261–1278. 36863689 10.1053/j.gastro.2023.02.005

