# Supplementary material for: MMR deficiency is frequent in colorectal carcinomas with diffuse SLFN11 immunostaining: clinicopathologic and molecular study of 31 cases identified among 3,300 tumors
Source: J Pathol Clin Res. 2025 Mar 19;11(2):e70025. doi: 10.1002/2056-4538.70025 (PMC11920882; doi:10.1002/2056-4538.70025)
Supplement: Supplementary file 1 — Supplementary materials and methods. [file CJP2-11-e70025-s004.pdf]

MMR deficiency is frequent in colorectal carcinomas with diffuse SLFN11 immunostaining: clinicopathologic and molecular study of 31 cases identified among 3,300 tumors

M Kaczorowski *et al.*, *J Pathol Clin Res*, https://doi.org/10.1002/2056-4538.70025

Supplementary materials and methods

IMMUNOHISTOCHEMICAL STAINING PROCEDURES

Antigen ID	Host and clonality	Clone	Vendor	Catalog #	Dilution	Procedure	Detection	Platform
SLFN11	mouse mAb	D-2	Santa Cruz	sc-515071	1:500	25' ER2, 20' inc	PRD	Leica Bond-Max
CDX2	rabbit mAb	EPR2764Y	Roche	760-4380	predilute	64' CC1, 36' inc	UV + amp	Ventana Benchmark Ultra
CK7	rabbit mAb	SP52	Roche	790-4462	predilute	64' CC1, 24' inc	UV + block	Ventana Benchmark Ultra
CK20	mouse mAb	Ks20.8	DAKO	M7019	1:250	16' protease 1, 32' inc	UV	Ventana Benchmark Ultra
β-catenin	mouse mAb	14	Roche	760-4242	predilute	64' CC1, 32' inc	UV + amp	Ventana Benchmark Ultra
p53	mouse mAb	DO-7	DAKO	M7001	1:2000	40' CC1, 60' inc	OV	Ventana Benchmark Ultra
MSH2	mouse mAb	G219-1129	Roche	760-4265	predilute	64' CC1, 120' inc	UV + amp	Ventana Benchmark Ultra
MSH6	mouse mAb	44	Roche	790-4455	predilute	64' CC1, 120' inc	UV + amp	Ventana Benchmark Ultra
MLH1	mouse mAb	M1	Roche	790-4535	predilute	64' CC1, 24' inc	OV	Ventana Benchmark Ultra
PMS2	rabbit mAb	EPR3947	Roche	760-4531	predilute	92' CC1, 32' inc	OV + amp	Ventana Benchmark Ultra

mAb: monoclonal antibody; inc: incubation with primary antibody; PRD: Leica Bond Polymer Refine Detection; OV: Ventana OptiView; UV: Ventana UltraView; amp: amplifier

MOLECULAR GENETIC STUDIES

1. Nucleic acid extraction

1.1 Extraction of tumor DNA for NGS and ddPCR and normal DNA from tumor-adjacent non-neoplastic tissues for mutation-specific PCR amplification and Sanger sequencing

DNA was extracted from multiple (3 to 10) formalin fixed paraffin embedded (FFPE) 5 μm tissue sections using Maxwell® RSC DNA Kit and Maxwell® RSC instrument (Promega, Madison, WI). The Qubit fluorometer and Qubit dsDNA BR Working Solution kit were used for the DNA quantification (Thermo Fisher Scientific, Waltham, MA).

1.2 Extraction of tumor DNA for MLH1 promoter hypermethylation analysis

DNA was extracted from 20 μm thick FFPE tissue section using a QIAsymphony DNA Mini Kit and automated extraction system (Qiagen, Hilden, Germany) according to the manufacturer's protocol (Purification of genomic DNA from FFPE tissue using the QIAamp DNA FFPE Tissue Kit and Deparaffinization Solution). DNA concentration and purity was examined by the NanoDrop ND-1000 spectrophotometer (Nano Drop Technologies LLC., Wilmington, DE) while integrity was evaluated by multiplex PCR amplification of control sequences as previously reported. (PMID: 14671650)

1.3 Extraction of DNA and RNA from peripheral blood for germline testing

Peripheral blood samples were collected into the EDTA- and the PAXgene Blood RNA-vacutainer tube for DNA and RNA extraction, respectively. Extractions were performed using either QIAsymphony DNA DSP Mini Kit (Qiagen) or PAXgene Blood RNA Kit (PreAnalytix-Qiagen) following manufacturer's protocols.

2. Tumor next generation sequencing (NGS) with 50 gene panel

The Ion Torrent™ (Life Technologies/Thermo Fisher Scientific, Waltham, MA) platform and 50 gene Ion AmpliSeq™ cancer hotspot (CH) panel were used as previously reported (PMID: 30760858). Data were extracted from the Ion Torrent Server using plugins available on Torrent Suite™ Software version 5.12.3 (Thermo Fisher Scientific). Sequences were aligned to human genome assembly GRCh37/hg19 University of California Santa Cruz version (https://www.genome.ucsc.edu/). Variant calling was performed using Variant Caller v5.12.3 compatible with the Integrative Genomics Viewer (Broad Institute, Cambridge, MA), a high-performance visualization tool for interactive exploration of large, integrated data sets. A threshold of ≤10% was used to define low allele frequency

The analysis employed publicly available bioinformatics tools and databases. wANNOVAR server (WGLAB, https://wannovar.wglab.org/) was used to annotate the functional consequences of mutation occurrence. The alterations were then analyzed using the Cancer Genome Interpreter (In silico saturation mutagenesis of cancer genes [doi: https://doi.org/10.1101/140475]) for their clinical utility or carcinogenic potential. Subsequently, databases such as ClinVar (NCBI NIH, https://www.ncbi.nlm.nih.gov/clinvar/) and OncoKB (PMID: 28890946 PMID: 37849038 https://www.oncokb.org/) were used to determine the pathogenicity of the variant. In the absence of information in the aforesaid databases, the VarSome (PMID: 30376034, https://varsome.com/) and Franklin (https://franklin.genoox.com) bioinformatics tools were incorporated. VarSome verdicts based on evaluation of multiple methods predicting mutation pathogenicity are listed in the sequence database (Supplemental data).

Mutation nomenclature was based on Human Genome Variation Society (https://www.hgvs.org/) recommendations. Gene names and symbols are as recommended by the GeneCards (https://www.genecards.org/). Reference sequences were obtained from the National Center for Biotechnology Information's reference sequence (NCBI's RefSeq) database (http://www.ncbi.nlm.nih.gov/RefSeq/).

3. Tumor NGS with a customized gene panel

The customized panel which covers 11 genes (*KRAS*, *BRCA1*, *BRCA2*, *CTNNB1*, *PIK3CA*, *POLE*, *TP53*, *MLH1*, *MSH2*, *MSH6*, *PMS2* and *POLD1*) was created using the Ion Ampliseq Designer tool (Thermo Fisher Scientific). The method enables detecting SNVs, small deletions and insertions of up to 15 nucleotides. Rearrangements involving one exon, or several exons were beyond the scope of this assay. Also, the detection of changes within homopolymers was limited. Moreover, changes occurring within primer binding sites may prevent detection of variants. The detection limit was ≥ 5% at a 99% confidence level.

The NGS has been performed using the Ion GeneStudio™ S5 Prime System. Gene libraries, prepared according to the manufacturer's instructions for Oncomine™ tumor specific panels (Cat. No. A35121) were sequenced using Ion Torrent technology (described in the 2nd section)

4. Analysis of MLH1 promoter methylation status

EZ DNA Methylation-Gold Kit (Zymo Research, Burlington, ON, Canada) was used for the bisulfite conversion of DNA samples. Subsequently the methylation-specific PCR targeting the *MLH1* promoter was performed and evaluated as previously reported (PMID: 11839573).

5. Droplet Digital™ PCR (ddPCR™) Microsatellite Instability (MSI) assay

The MSI status was assessed by using ddPCR MSI Kit and QX200 Droplet Reader (Bio-RAD, Hercules, CA) following the manufacturer's protocol. ddPCR MSI Kit included 3 assays/5 markers (assay 1: *BAT-25*, *BAT-26*; assay 2: *NR-21*, *NR-24*; assay 3: *MONO-27*) highly sensitive and specific for the detection of mismatch repair (MMR) defects in microsatellite repeats. The results were interpreted as recommended by the protocol. Namely, cases with none of the five microsatellite markers altered were classified as microsatellite stable (MSS) while cases with ≥40% of microsatellite markers altered (≥2 altered markers out of 5) were classified as microsatellite instability-high (MSI-H).

6. MLH1, MSH2, MSH6 and PMS2 germline testing using DNA and RNA from peripheral blood and Sanger sequencing

MMR gene germline testing was performed using Sanger sequencing as described previously. (PMID: 26779764) Coding sequence of *MLH1*, *MSH2* and *MSH6* and exon-intron junctions were PCR amplified using genomic DNA. *PMS2* coding sequence was RT-PCR amplified to avoid cross-amplification of *PMS2* pseudogenes. PCR reaction consisted of 11µl of Fast Start PCR Master Mix (Qiagen), 1.8µl of 5µM forward and reverse primer mix, 8µl of PCR grade water and 1.3µl of template DNA (50-100 ng/µl). Following initial denaturation at 95°C for 10 min., 40 PCR cycles (95°C/ 60s, 60°C/60s, 72°C/60s) were performed with final extension at 72°C for 7 min. PCR products were purified with the AMPure magnetic particles (Agencourt Bioscience Corporation, A Beckman Coulter Company, Beverly, MA) and added to the sequencing reactions using the Big Dye Terminator Sequencing Kit (Applied Biosystems, Foster City, CA) and cleaned with the CleanSEQ magnetic particles (Agencourt Bioscience

Corporation). Electrophoretograms were obtained on an automated genetic analyzer ABI Prism 3130xl (Applied Biosystems, Waltham, MA) at a constant voltage of 13.2 kV for 20 minutes. The analyzed sequences were compared to the appropriate reference sequences.

7. Germline testing using mutation-specific PCR amplification and Sanger sequencing

DNA samples from non-neoplastic tissues adjacent to MLH1 (n=1), MSH2 (n=1), and MSH6 (n=1) mutant tumors (identified by a custom gene NGS panel) were germline tested using mutation-specific PCR amplification and Sanger sequencing. DNA templates were PCR amplified using KAPA SYBR® FAST DNA polymerase (Roche, Basel, Switzerland) and mutation-specific primers in the Veriti™ Thermal Cycler 96-well Fast (Applied Biosystems™, Foster City, CA, USA). Forty-cycle PCR amplification consisted of 3s denaturation (95°C), 20s primer annealing (MLH1 59°C, MSH2/MSH6 60°C) and 30s elongation (72°C) and was folowed by the final 3 min elongation at 72°C. Products were purified using Exonuclease I and FastAP Thermosensitive Alkaline Phosphatase (Thermo Scientific, Waltham, MA). The sequencing reactions were performed on the Veriti™ Thermal Cycler (Applied Biosystems) using BigDye™ Terminator v3.1 Cycle Sequencing Kit (Thermo Scientific) and following conditions: first stage at 37°C for 15 min., second stage at 85°C for 15 min. Samples purified with BigDye XTerminator™ Purification Kit were suspended in Hi-Di™ Formamide and analyzed on the 3500 Series Genetic Analyzer or SeqStudio™ Genetic Analyzer (Applied Biosystems). Generated chromatograms were viewed by Chromas (https://technelysium.com.au/wp/chromas/) and compared to reference sequences using BLAST (https://blast.ncbi.nlm.nih.gov/Blast.cgi).

7.1 Mutation-specific primers

MLH1 c.775_776insA (p.Leu259Tyrfs*48), forward primer TGTGAGGATAAAACCCTAGCC (annealing temperature [atemp.] 58.2°C), reverse primer: aaccaaactttgccatgagg (atemp. 60°C); MSH2 c.1147C>T (p.Arg383Ter), forward primer: cccagcagattcaagctttt (atemp. 59.5°C), reverse primer: ggacagcacattgccaagta (atemp. 59.7°C)

MSH6 c.362del (p.Arg121fs), forward primer: tccttttggcaacagTTGTG (atemp. 59.7°C), reverse primer: caaacacacacacatggcagt (atemp. 60.5°C).

8. The selective adaptor ligation selective amplification methylation sensitive-multiplex ligation probe amplification (SALSA® MS-MLPA®)

The SALSA MS-MLPA kits P003, P072, P008, and ME011 (MRC-Holland, Amsterdam, the Netherlands) were used to examine large deletions, duplications, and aberrant methylation in the *MLH1*, *MSH2*, *MSH6*, and *PMS2* genes according to the manufacturer's instructions. The PCR products were separated by capillary electrophoresis using ABI 3130XL Genetic Analyzer (Applied Biosystems), and the output data were analyzed with Coffalyser.net software (MRC-Holland).
